# Haematoma Auris No Evidence of Insanity or of Impending Insanity

**Published:** 1890-09

**Authors:** Hugh Hagan

**Affiliations:** Atlanta, Ga.


### HAEMATOMA AURIS NO EVIDENCE OF INSANITY OR OF IMPENDING INSANITY.

BY HUGH HAGAN, M. D.. ATLANTA, GA.

For many years the question of relationship between haematoma auris and insanity, being close, or sanguineous, if I may use the expression, has been accepted as an alienistic fact.

As the literature on the subject is quite unsatisfactory, although plentiful, yet for some years, nothing new has been produced, and in consequence, the old ideas have become fixed, and few investigations made.

Dr. Hun, in his interesting and scientific works on the subject, states, "that in his experience 24 cases occurred, of which 23 were in men and one in woman;" and later on, that either persons with haematoma auris are insane, or are to be looked upon as patients, and will sooner or later prove insane."

Most other authors accept Dr. Hun's views, with the notable exceptions of Bryant in England, and the older Gross in our country. These two authorities accept Dr. Hun's views so far as to acknowledge a relationship and dependency, but do not go so far as to state that insanity is present, or will be.

Prof. Griesinger in his (Psychische Krankheiten,) states, "that the condition under consideration is not peculiar to insanity per se, but when occurring in the insane, whether in progressive paralysis, or any other form, is due to some extraneous cause, such as blows from attendants, violence upon their own persons, etc., or from some general systemic condition, as haemophilia and as a sequel to the exhaustive diseases, which produce like conditions in the sane.

We all well know that in the early part of this century, and further back in the past, before the humane efforts of Conolly and Pinel were crowned with success, the insane were looked upon as being possessed of many devils, witches, almost beasts. When such means as the ducking stool, the lash, the stake, straight jacket, and what not, were the means used to free the unfortunates of their unholy tenants, or the community of the possessed; too often the latter; when the poor unfortunates were consigned to their dungeons and inhuman keepers.

In the past few years both in this country and in Europe, it has been my lot to see and examine, or be present at the examination of no less than 2,500 insane persons, and in this large number of insane, comprising all forms of insanity, there was not one case of haematoma auris. In the mean time I have seen two cases, one a man and one a woman, both perfectly sane, and of healthy genealogy, and both cases were due to direct traumatism, one, the man, by a blow from the fist of an antagonist, and the woman, from a blow received by a fall.

In last month's Southern Medical Record, I saw the account of a case reported by my friend Dr. Chas. M. Shields, of Richmond, Va., in a man, who was injured by falling and striking his head. This patient was sane. When we stop to think we will notice that the condition is of much rarer occurrence than it was wont to be, and considering the statement of Griesinger, we shall see the reason. In section 194, p. 447-448 of his work on the diseases of the mind, published in Braunschweig, he says, "among the frequent local affections of the insane, is first to be noticed, the much discussed disease of the external ear, generally known as haematoma auris. The skin of the auricle becomes glossy, stretched, and shows an indistinct fluctuation; the whole external ear becomes thicker, bluish red, hot and painful. On incising the tumor, one finds a cavity filled with an imperfect bloody coagulum. Occasionally the cavity empties itself by spontaneous rupture. By closer examination or inspection, one finds a sanguineous extravasation under the perichordium, which separates it from the cartilage. Generally in a few weeks the swelling and redness lessen, and there remains a perceptible thickness of the collapsed sack walls.

According to some authorities a new production of cartilage is found, which is followed by shrinking, leaving a deformity of the auricle. About the origin of this condition there has been much discussion, and is already much more literature on the subject than is deserved, or necessary. While a number of authorities consider its pontaneous process, a haemorrhagic chondritis, analagous to pachy meningitis, others as a result of cerebral congestion, while yet others declare it to be a purely external condition, caused through traumatism, either in the form of blows on the head, from contact with the walls and bedposts, and partly through boxing and pulling the ears by inhuman and powerful attendants. The view that this condition is produced by a purely accidental, and traumatic origin, in the vast majority of cases has in later times been well substantiated, and especially by Gudden, and it is by far the most probable and possible.

The ear affection comes most exclusively in male patients, who have male attendants, and always in the asylums; it is most frequent on the left ear, (because this is the most accessible to the right hand of the attendant). It arises very quickly, and one often finds the imprints of the finger nails, and the affection can be prevented in well regulated asylums by constant watching the attendants."

I have quoted Greisinger thus fully, because here we have a complete survey of the subject. Now to compare Hun's cases, we find that of the 24 patients 23 were males, and to this we can but ascribe the rough handling as the cause, for are not women insane as well as men? Then, too, by constant watch we find the total prevention of the condition by the improved and humane treatment to which the insane are subjected—the condition is very rare, almost unknown.

There is no acknowledged difference between the pathology of the disease in the sane and insane. If the condition were due to insanity per se would it not be relatively as frequent in private as in the asylum? And lastly, how can one reconcile the disparity existing between the common idea, that the condition is confined to the insame, when of 2,500 insane not one case was recorded, while the writer has personally attended two cases in the sane, and cites one recent case.

